# The Role of Telemedicine in Improving Hypertension Management Outcomes: A Systematic Review

**DOI:** 10.7759/cureus.74090

**Published:** 2024-11-20

**Authors:** Salma Hassan M Ali, Areij Awad Osman Mohamed, Hanady ME M Osman, Mohamed Elsayed Abdelrahman Ibrahim, Mohamed Ahmed Hassan Mukhtar, Fatima Hadab Ahmed Mohamed, Ali Hadi M Alhajri

**Affiliations:** 1 Obstetrics and Gynecology, Najran Armed Forces Hospital, Najran, SAU; 2 Internal Medicine, Sudan Heart Center, Khartoum, SDN; 3 Quality and Patient Safety, Najran Armed Forces Hospital, Najran, SAU; 4 Internal Medicine, Najran Armed Forces Hospital, Najran, SAU; 5 Emergency Medicine, Najran Armed Forces Hospital, Najran, SAU; 6 Geriatric Medicine, University Hospitals of North Midlands NHS Trust, Newcastle, GBR

**Keywords:** high blood pressure, hypertension, remote monitoring, telehealth, telemedicine

## Abstract

Telehealth has been proven to be effective in a variety of healthcare settings and has enhanced patient utilization of healthcare services. Little is known about the use of telehealth in the treatment of hypertension. This study aimed to categorize and identify data related to various telehealth technologies and intervention types used in the management of hypertension. The Preferred Reporting Items for Systematic Reviews and Meta-Analyses guidelines were used to search the literature based on predetermined inclusion and exclusion criteria. These databases contained 1,483 relevant articles, which were screened for duplication using Endnote software. After a careful full-text article evaluation, only 42 of these articles were found to be relevant. The Newcastle-Ottawa Scale was used to assess the risk of bias in each included study. The majority of studies (23.8%) were conducted in urban areas (33.3%), were from the United States, and used a quantitative study approach (69%), according to the proportions of studies displaying different patterns over the past 10 years. Telemonitoring and teleconsultation are the two most used telehealth techniques for managing hypertension. Asynchronous telehealth is quickly becoming the most popular technique for controlling hypertension. In hypertension management, telehealth refers to the use of communication technologies to remotely monitor and regulate blood pressure as well as offer medical advice and counseling.

## Introduction and background

The practice of using medical data transferred electronically between locations to enhance an individual’s clinical health state is usually referred to as telemedicine [1]. Telemedicine encompasses an expanding range of services and applications that use wireless gadgets, smartphones, the Internet, two-way video, calls, email, and other telecommunications technologies [2].

About 40 years ago, telemedicine was first implemented in hospitals to care for patients in faraway locations [3]. Since then, its use has grown significantly. Telemedicine is becoming an essential component of healthcare and is increasingly being incorporated into the everyday activities of hospitals, particular departments, home health services, private physician offices, and consumers’ homes and workplaces [4]. However, there are still significant obstacles preventing its widespread adoption in everyday practice, including inadequate implementation, low payment levels, and a lack of physician support [5]. According to the latest Health Information Systems Technologies report, the total number of patients utilizing telemedicine services was expected to increase from roughly 350,000 in 2013 to 7 million in 2018 [6].

Telemedicine has a beneficial impact on all areas of medicine by providing patients and medical professionals with a tool to enhance disease management. Hence, the terms telemedicine and telehealth, which refer to a broad spectrum of remote medical services, have increasingly come to be used interchangeably [7]. Among other applications, telehealth includes patient consultations through video conferencing, still image delivery, patient websites, remote vital sign monitoring (also known as telemonitoring), continuing healthcare education, consumer-focused mobile apps, and nursing contact centers [8].

The potential of telemedicine in improving outcomes for patients with hypertension has been supported by various pilot studies and clinical trials [9]. These studies suggest that telemedicine can lead to improved blood pressure control, increased patient satisfaction, and reduced healthcare costs. However, while these results are promising, they are not without challenges. Factors such as technology accessibility, data privacy concerns, and the digital literacy of both patients and healthcare providers can impact the effectiveness and scalability of telemedicine services [10]. Additionally, differences in the implementation of telemedicine across various healthcare settings and regions can affect outcomes and create disparities in care.

This systematic review aims to consolidate and evaluate existing evidence on the role of telemedicine in improving hypertension management outcomes. By analyzing data from studies that explore telemedicine’s effectiveness, patient adherence, cost-efficiency, and impact on healthcare delivery, this review seeks to provide a comprehensive overview of its benefits and limitations. The findings will contribute to a better understanding of how telemedicine can be optimized to support hypertension management, ultimately informing future strategies and policies that harness technology for improved patient care.

## Review

Methodology

This systematic review followed the recommendations of the Preferred Reporting Items for Systemic Reviews and Meta-Analyses (PRISMA) guidelines [11].

Search Strategy

We searched five different databases to identify published research in English without considering the publishing timeline. Additionally, we searched these databases to identify any previous or ongoing systematic reviews on the subject. Results from many databases were combined and duplicates were eliminated using Endnote software. Table 1 lists the databases and search techniques that were employed.

**Table 1:** Search string used for different databases.

Database	Search string
Scopus	(Telemedicine OR “telehealth” OR “remote consultation” OR “virtual care”) AND (Hypertension OR “high blood pressure”) AND (management OR treatment OR “clinical outcomes” OR "patient outcomes" OR “blood pressure control”)
Web of Science	(Telemedicine OR telehealth OR “remote consultation” OR “virtual care”) AND (Hypertension OR “high blood pressure”) AND (management OR treatment OR “clinical outcomes” OR “patient outcomes” OR “blood pressure control”)
PubMed/EMBASE	(“Telemedicine”[Mesh] OR telemedicine OR telehealth OR “remote consultation” OR “virtual care”) AND (“Hypertension”[Mesh] OR hypertension OR “high blood pressure”) AND (management OR treatment OR “clinical outcomes” OR “patient outcomes” OR “blood pressure control”)
Google Scholar	(Telemedicine OR telehealth OR “remote consultation” OR “virtual care”) AND (Hypertension OR “high blood pressure”) AND (management OR treatment OR “clinical outcomes” OR “patient outcomes” OR “blood pressure control”)
Cochrane Library	(Telemedicine OR telehealth OR “remote consultation” OR “virtual care”) AND (Hypertension OR “high blood pressure”) AND (management OR treatment OR “clinical outcomes” OR “patient outcomes” OR “blood pressure control”)

Study Selection

Duplicates were removed throughout the article extraction process, and each article was extracted and stored in the Endnote library (ENDNOTE X9). The selection of the included studies was done by two different reviewers. Reviewer 1 (SHMA) independently evaluated abstracts and titles two times, while reviewer 2 (MAHM) recognized studies based on the data and resolved any disputes over any included study. The publications were chosen for inclusion based on the inclusion and exclusion criteria following a comprehensive analysis by reviewers that determined whether they offered the relevant data for the systematic review (Table 2).

**Table 2:** Inclusion and exclusion criteria.

Questions	Inclusion criteria	Exclusion criteria
Type of studies	Randomized controlled trials, cohort studies, cross-sectional studies, and qualitative studies	Case reports, editorials, opinion pieces, conference abstracts without full text, and non-peer-reviewed articles
Population	Studies involving adult patients diagnosed with hypertension	Studies focusing on pediatric populations or those not specifically targeting hypertensive patients
Intervention	Studies examining telemedicine or telehealth interventions, including remote monitoring, virtual consultations, or digital health technologies used for hypertension management	Studies not involving telemedicine or telehealth as the primary intervention or focussing on unrelated digital tools
Outcomes	Studies reporting on hypertension management outcomes such as blood pressure control, patient adherence, clinical outcomes, or patient satisfaction	Studies not reporting on specific clinical or patient outcomes (e.g., studies focusing solely on technical aspects without patient outcomes)
Study language	Studies published in English	Studies published in any other language

Data and records were extracted and stored using a Microsoft® Excel Spreadsheet (Microsoft, Inc., Redmond, WA, USA).

Risk of Bias Assessment

The Newcastle-Ottawa Scale (NOS) was used to evaluate the risk of bias in the included studies. Based on selection process bias, intervention bias, departure from intervention bias, missing data bias, outcome bias, and results bias, studies were rated as low, moderate, or high. Preference for selection was determined using the inclusion and exclusion criteria. Performance bias was evaluated by describing a control arm and taking allocation concealment into account. Data management, selective reporting, biased reporting, and complete industrial sponsorship received varying ranks. Reviewers examined reporting consistency and eligibility restrictions throughout multiple sessions. Any differences in the reviewers’ scores were taken into consideration when a second reviewer selected the research.

Results

Search Results

**Figure 1:**
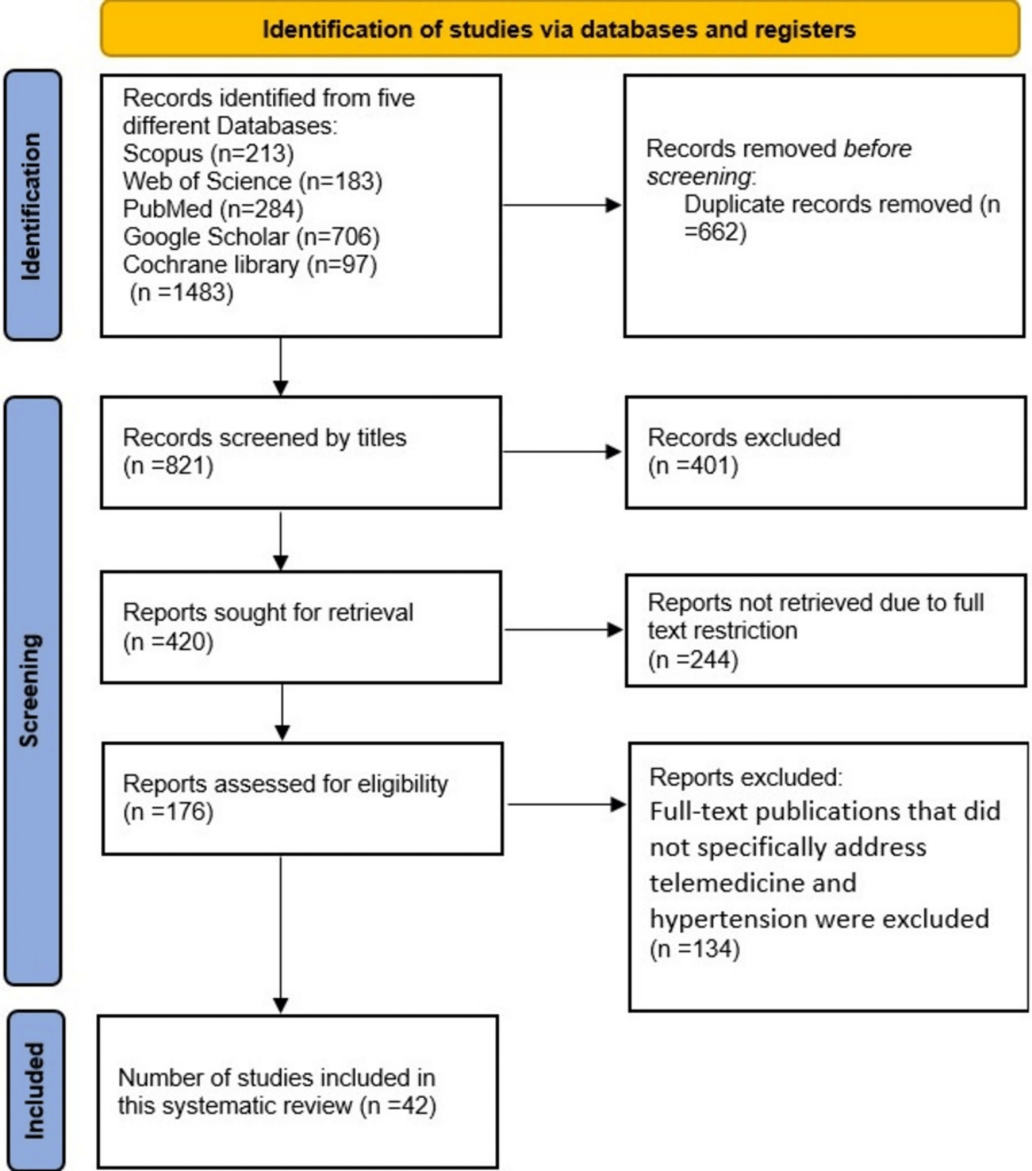
Preferred Reporting Items for Systemic Reviews and Meta-Analyses flowchart of study selection.

Following the study selection criteria, we identified 1,483 studies, of which 662 duplicates were removed. We identified 821 studies and assessed them based on titles, with 401 studies found irrelevant and excluded. The remaining 420 studies were searched to determine full-text availability. In total, 244 studies could not be retrieved and were excluded from the study. Overall, 176 full-text articles were assessed for eligibility, of which 134 either did not focus specifically on hypertension or telehealth approaches and were excluded. Finally, 42 studies were found to be eligible and included in this systematic review (Figure 1).

Risk of Bias Assessment

The NOS was used to assess the risk of bias. Overall, 14 of the 42 studies had a low-risk bias, 27 had a moderate-risk bias, and one had a high-risk bias. The selection of the controls in some studies was one of the methodological flaws. Additionally, no study revealed how controls and patients were blinded to exposure, which could have resulted in measurement bias (Table 3).

**Table 3:** Risk of bias assessment of the studies included in the systematic review. Rating scale: 7 to 9 stars = low risk of bias; 4 to 6 stars = moderate risk of bias; 0 to 3 stars = high risk of bias. Selection: (1) Is the definition sufficient? (2) Is the representativeness of the case acceptable? (3) Selection of controls (hospital or community). (4) Definition of controls. Comparability: (1) Comparability of cases and controls based on design or analysis. Exposure: (1) Determining exposure. (2) Calculation of controls and cases using the same methodology. (3) Non-response rate. A study gets one star (★) for each numbered item in the selection and exhibit categories. Two stars (★★) are assigned for comparability.

Study	Selection				Comparability	Exposure		
	1	2	3	4	1	1	2	3
Teo et al. [12]	★	★			★★	★		★
Silveira et al. [13]	★	★			★	★	★	
Saleh et al. [14]	★	★				★	★	★
Grant et al. [15]	★	★	★		★★	★	★	★
Shaw et al. [16]	★	★			★★	★	★	★
Cimini et al. [17]	★	★			★	★	★	★
Sin et al. [18]	★	★			★★		★	★
Calderón-Anyosa et al. [19]	★	★		★	★★	★	★	★
Ye et al. [20]	★	★	★		★		★	★
Nau et al. [21]	★	★			★★	★	★	★
Abdullah et al. [22]	★	★					★	
Cottrell et al. [23]	★	★				★	★	★
Kassavou et al. [24]	★	★	★		★★	★	★	★
Andersson et al. [25]	★	★	★		★	★	★	
Chew et al. [26]	★	★				★	★	
McManus et al. [27]	★	★			★★	★	★	★
Davoudi et al. [28]	★	★			★	★	★	★
Debon et al. [29]	★	★		★		★	★	★
Peters et al. [30]	★	★	★		★★	★	★	★
Marcolino et al. [31]	★	★			★★	★	★	★
Lee et al. [32]	★	★		★	★	★	★	★
Manusov et al. [33]	★	★				★	★	★
Nurakysh et al. [34]	★	★	★			★	★	★
Ju et al. [35]	★	★	★			★	★	★
Cottrell et al. [36]	★	★				★	★	★
Buis et al. [37]	★	★			★★	★	★	★
Leon et al. [38]	★	★			★	★	★	★
Doocy et al. [39]	★	★		★			★	★
Jindal et al. [40]	★	★	★		★★		★	★
Chen et al. [41]	★	★			★★		★	★
De Luca et al. [42]	★	★		★	★		★	★
Ashjian et al. [43]	★	★			★★	★	★	★
Levine et al. [44]	★	★	★		★★	★	★	★
Ma et al. [45]	★	★	★		★★	★	★	★
Fisher et al. [46]	★	★			★		★	
Parker et al. [47]	★	★			★	★	★	★
Koopman et al. [48]	★	★			★★		★	★
Buis et al. [49]	★	★	★		★★	★	★	
Santos et al. [50]	★	★			★★	★		
Vedanthan et al. [51]	★	★		★	★	★	★	
Naqvi et al. [52]	★	★			★★	★	★	
Barsky et al. [53]	★	★	★		★		★	★

The studies included in this systematic review had low-quality evidence, according to GRADEpro GDT. The low quality of the evidence was mostly caused by the uneven nature of the research and the inclusion of observational studies (case-control), which raises the risk of bias due to the inability to randomize exposure.

Characteristics of Included Studies

The majority of the studies in this review were published after 2013. The United States reported the majority of the studies (N = 10), followed by Brazil (N = 5) and the United Kingdom (N = 3). The majority of the investigations included in this review employed a quantitative strategy, followed by mixed methodologies and qualitative approaches, depending on the type of research. Concerning geographic contexts, the majority of the research was conducted in urban areas, followed by rural areas. From 2013 to 2024, the number of investigations documenting the use of telemedicine in the management of hypertension tended to fluctuate. The years 2019 and 2022 witnessed the greatest number of published studies (N = 7) (Table 4).

**Table 4:** Characteristics of the included studies. CDSS: clinical decision support system; RCT: randomized controlled trials; TeleHAS: tele–Hipertensão Arterial Sistêmica; DSS: decision support system; CHW: community health worker; TM: telemonitoring; BP: blood pressure; TASC: telehealth after stroke care; nRCT: non-randomized controlled trial; UniMóvil: a mobile health clinic providing primary care; BPMED: blood pressure medication; DESIRE: decision support and integrated record keeping

Citation	Publishing year	Country	Study design	Type of population	Intervention	Key findings
Teo et al. [12]	2024	Singapore	Mixed methods	Urban	Blood pressure monitoring at home	Compared to standard treatment, telemonitoring combined with teleconsultation was more economical and improved blood pressure control. In addition, telemonitoring intervention patients expressed greater motivation and satisfaction with their treatment
Silveira et al. [13]	2019	Brazil	Mixed methods	Urban	TeleHAS	In a middle-income country’s primary healthcare system, a CDSS designed to help manage hypertensive patients was practical, had high user satisfaction, and had the potential to increase adherence to evidence-based therapies
Saleh et al. [14]	2018	Lebanon	Mixed methods	Rural	SMS	A more specialized strategy is required for older, illiterate, and jobless people, even if SMS-based treatments for those with diabetes and/or hypertension were generally well received by those residing in remote regions including Palestinian refugees in Lebanon
Grant et al. [15]	2019	UK	RCT	Rural and urban	Text message	The realities of modern UK healthcare meant that a written approach to monitoring oneself could be more easily incorporated into present workflows, even though mHealth’s telemonitoring makes communication and comfort easier. All hypertensive patients should have access to self-monitoring
Shaw et al. [16]	2013	USA	Mixed methods	Rural and urban	Nurse-delivered self-management phone	Key enablers and impediments to organizational change, preparedness, and successful execution were identified by the organizational change model. Understanding the requirements and difficulties of implementing an intervention was made possible by the study
Cimini et al. [17]	2022	Brazil	Multimethodological approach	Not reported	A digital solution with a DSS for CHWs	The number of primary care consultations for people with diabetes and hypertension fell sharply during the COVID-19 epidemic. It has been demonstrated that a DSS of CHW is practical, helpful, and simple to integrate into their daily tasks
Sin et al. [18]	2020	Singapore	Cross-sectional	Urban	Telemonitoring	Among the patients, somewhat more than half were open to using TM. Before using TM in primary care, it is necessary to address factors including age, ethnicity, technological understanding, beliefs, and the patients’ prior technology use
Calderón-Anyosa et al. [19]	2023	Peru	RCT	Urban	SMS-based home BP tele-monitoring system	The study concluded that, when used in combination with primary care facilities, an SMS-based home blood pressure monitoring system effectively lowers diastolic blood pressure. The results of the study constitute a significant alternative for the management of hypertension and one of the first such interventions in our environment
Ye et al. [20]	2022	South America	Retrospective cohort study	Urban	Video and telephone	Using telemedicine visits more frequently is linked to worse results on managing high blood pressure performance score. However, when blood pressure is documented, using telemedicine visits might not have a negative effect on blood pressure control
Nau et al. [21]	2021	Australia	Pilot study	Urban	Videos, web-based education, and text message	Using additional mobile health initiatives, lifestyle changes for managing the symptoms of elevated blood pressure within a hectic primary care setting can be improved. To find tactics that can be included in standard care and result in high patient participation, more intervention improvement and formative assessment are needed
Abdullah et al. [22]	2016	Malaysia	Qualitative study	Urban	A blood pressure telemonitoring service	Although patients deemed the primary care blood pressure telemonitoring service to be user-friendly, they required assistance in deciphering the meaning of the monitored blood pressure values. To increase patient acceptance of a hypertension telemonitoring service, several problems must be addressed during implementation
Cottrell et al. [23]	2015	UK	Evaluation study	Not reported	Text messages	When individuals were carefully chosen for the protocol, professional users were conversant with the platform, the program discussed an issue with the prior service delivery that users had identified, and users actively pursued clinical objectives, satisfaction with AIM seemed to be at its highest
Kassavou et al. [24]	2019	UK	Descriptive cross-sectional	Not reported	Highly tailored text and voice message	As a supplement to primary care, customized computerized text and audio interventions are workable means of enhancing drug adherence
Andersson et al. [25]	2021	Sweden	Qualitative study	Urban and Rural	Interactive web-based system	As a result of using the system, patients became more knowledgeable about their blood pressure and took an active role in its treatment. The professionals found the system to be a helpful tool for communicating about blood pressure and lifestyle when it was used as intended. Professionals and patients discussed an appointment on more equal terms
Chew et al. [26]	2023	Singapore	Qualitative study	Urban	A remote blood pressure monitoring program	For telemedicine to be implemented successfully, managing trust connections is crucial. Enhancing the efficacy and caliber of care can be achieved by ensuring that trust-building is integrated into the design of telemedicine interventions
McManus et al. [27]	2021	UK	RCT	Not reported	Home and online management	After a year, the blood pressure digital intervention, which uses self-monitored blood pressure to manage hypertension, improved systolic blood pressure control compared to standard care at a minimal incremental cost. Integration into medical processes and consideration of those who are electronically excluded are necessary to be implemented in primary care settings
Davoudi et al. [28]	2020	USA	Secondary analysis of RCTs	Not reported	Automated text messaging	Patients using an automated text messaging network for remote blood pressure monitoring showed distinct interaction patterns. The only communication style linked to reaching the desired blood pressure was minimalistic. Future automated texting conversations and intervention designs to improve blood pressure control may benefit from the identification and comprehension of interaction phenotypes
Debon et al. [29]	2020	Brazil	nRCT	Not reported	Use of a mobile health app	Patients with AH receiving therapy at the FHS reported improved health outcomes while using mHealth apps, particularly when paired with medical data. In the FHS context, technology utilization is promoting and facilitating improved health outcomes
Peters et al. [30]	2017	USA	Qualitative study	Not reported	Phone call and SMS text messaging	For patients who have high baseline blood pressure despite prior treatment, EpxHypertension offers a practical way to manage hypertension. The viability of using EpxHypertension in a primary care context without the use of cellphones or Bluetooth-enabled blood pressure monitors is demonstrated by this community implementation study
Marcolino et al. [31]	2021	Brazil	Mixed methods	Rural and urban	Teleconsultation	The CDSS had the ability to increase adherence to evidence-based procedures and was useful in primary care settings in low-income areas. It also had good user satisfaction
Lee et al. [32]	2022	USA	Cohort study	Urban	Remote blood pressure monitoring	The results imply that a combination of office-based and remote management was successfully prompted by EHR notifications for increased blood pressure during remote monitoring. Additionally, it was typical for the care plan to stay the same, which may indicate that better clinical support and more sophisticated alarms are needed
Manusov et al. [33]	2019	USA	Retrospective study	Rural	UniMóvil, a mobile health clinic	Mobile clinics improve access and treat diseases that are quite common in Colonias. The information gathered can be utilized to target care, address quality of life and chronic illness, and guide research in underserved, high-need areas
Nurakysh et al. [34]	2022	Kazakhstan	Multicenter RCT	Not reported	Mobile application “MyTherapy”	The efficiency of using the “MyTherapy” mobile application to increase patient adherence was demonstrated by an investigation of adherence among patients with persistent arterial hypertension receiving primary care in Almaty, Kazakhstan. To determine whether broader adoption of digital technologies in healthcare is feasible, more research is needed to evaluate the positive effects of their use
Ju et al. [35]	2022	South Korea	Pilot study	Not reported	A mobile self-management healthcare app	Chronic condition treatment is improved when primary care facilities and a mobile managing oneself healthcare application with human coaching are combined
Cottrell et al. [36]	2015	UK	Evaluation study	Not reported	Text messaging (“Florence”)	There were issues found, despite the fact that basic telehealth might be a valid method for identifying and tracking hypertension in responding patient users. This is especially true if blood pressure surveillance is not feasible or is refused
Buis et al. [37]	2017	USA	Unblinded RCT	Urban	Automated text message	For African Americans with high blood pressure that is uncontrolled, using reminders for texts to increase adherence to medication is a practical and acceptable strategy. Even though there were no statistically significant differences in blood pressure or actual medication adherence between BPMED as well as usual care controls, trends of improvement across the BPMED condition indicate that text-based medication reminders might be effective, and fully powered studies with longer-term monitoring are necessary
Leon et al. [38]	2015	South Africa	RCT	Not reported	SMS	Even for patients who have their own reminder systems in place, adherence support for the treatment of high blood pressure via SMS text messaging on the patient’s personal phone was considered to be appropriate, pertinent, and beneficial
Doocy et al. [39]	2017	Lebanon	Cohort study	Urban	A mobile health app	The findings of the research on a mHealth application in 10 PHC institutions in Lebanon suggest that the app may enhance care quality and adherence to protocols
Jindal et al. [40]	2018	India	Not reported	Rural	Smartphone application (mWellcare)	Nurses working in primary care settings in India will use a pilot-tested mWellcare intervention, which is a mHealth system with key components, including evidence-based CDS, long-term health data, integrated treatment for chronic conditions, and a computer-generated short messaging assistance to reinforce adherence to medication intake and follow-up visits
Chen et al. [41]	2023	China	Longitudinal study	Urban	Online health management	Blood pressure control in hypertensive individuals is significantly and sustainably impacted by internet-based health management
De Luca et al. [42]	2021	Europe	User-centered approach	Not reported	Integrated management hypertension	The goal of a technologically enabled holistic approach to blood pressure is to educate the public on how to avoid hypertension, maintain an active lifestyle, and receive thorough, individualized treatment in close coordination with medical specialists
Ashjian et al. [43]	2019	USA	Observational study	Not reported	An interactive voice response (IVR)	Within our healthcare system, the IVR Mobile You High Blood Pressure Program is a novel use of digital technology. One of the obstacles to patient involvement that the pharmacist noticed was the availability of a validated home cardiac monitor
Levine et al. [44]	2018	USA	Retrospective cohort study	Not reported	Virtual visits	Participation in virtual visits was linked to lower in-office primary care usage and comparable blood pressure control among individuals with moderately well-controlled hypertension
Ma et al. [45]	2022	China	RCT	Urban	Smartphone-enhanced nurse-facilitated self-care intervention	Chinese hypertensive individuals in two communities may benefit from the phone-enhanced nurse-facilitated taking care of oneself intervention in terms of blood pressure, anthropometric measurements, and self-care. Future research can look at its long-term impact across various populations of hypertensive patients
Fisher et al. [46]	2019	USA	Prospective cohort study	Not reported	A home-based BP control program	An inventive approach to managing hypertension may be suggested by the efficient, quick, and effective control that non-physicians can provide with a home-based blood pressure control program
Parker et al., [47]	2018	UK	Prospective cohort study	Not reported	Text based tele-monitoring system	Patients who self-report their blood pressure utilizing telemonitoring have a preference for end-digits for 0 numbers and specific values for readings that are somewhat below the alert threshold. The percentage of impacted readings is minimal, though, and is not expected to be clinically significant
Koopman et al. [48]	2014	South America	Qualitative study	Not reported	Home blood pressure tele-monitoring	Effective implementations will be made possible by a thorough analysis of workflow and data flow, as is the case with many technology interventions
Buis et al., [49]	2020	USA	Pilot study	Urban and rural	BPTrack	The findings show that treating patients in primary care with hypertension that is uncontrolled with a pharmacy-led mHealth strategy that encourages home blood pressure tracking and clinical pharmacy treatment of hypertension can lower blood pressure
Santos et al. [50]	2013	Brazil	Pilot study	Rural	Education program	Following the CEP, adherence to a low-sodium diet and hypertension medication improved. This telehealth approach appears to have a beneficial effect on hypertension patients
Vedanthan et al. [51]	2015	Kenya	Cross-sectional study	Rural	Tablet-based DESIRE tool	The DESIRE tool’s deployment in western Kenya was hampered by unresolved usability and feasibility difficulties that were discovered throughout this iterative, inclusive human-centered design approach
Naqvi et al. [52]	2022	USA	RCT	Urban	Telehealth After Stroke Care	A definitive randomized study should be conducted to evaluate the potential of home blood pressure telemonitoring to improve the management of hypertension in an underserved context and enhance post-acute stroke care
Barsky et al. [53]	2019	Canada South Africa	Mixed methods	Rural	SMS text messaging-based system	The study’s technical aspects went according to schedule, and participants experienced support in managing their condition thanks to the mobile health program and SMS text messaging. Troubleshooting was used to resolve technological concerns

Discussion

The purpose of this study was to gather data on the various telehealth technologies and intervention types used in primary healthcare for the management of hypertension. The final synthesis comprised a total of 42 pertinent articles. This study was a systematic review that described how telehealth is used in primary practice to address hypertension. The majority of the articles, which were mostly conducted in urban areas, were from the United States, Brazil, and the United Kingdom. According to research methodologies, quantitative methods were used to analyze the majority of publications.

Our findings indicate that telemonitoring accounts for 69% of telemedicine management strategies. Vital sign transmission and remote monitoring should be a part of telemedicine in the treatment of hypertension [54]. Telemonitoring might work better than standard care in the short to medium term [55]. With minimal effect on physician burden, remote monitoring for high blood pressure can be implemented widely for primary healthcare [56]. Through better patient outcomes and lower healthcare expenditures, telemonitoring may enhance primary care treatment for cardiovascular disease [57,58]. According to a literature review, practically all of the research found that telemonitoring might lower individuals’ blood pressure [59].

Blood pressure monitors and other remote patient monitoring devices are frequently used in telehealth programs for hypertension management to track patients’ blood pressure values. A team-based care strategy is usually used in these interventions, with nurses, pharmacists, physicians, and other medical professionals working together to make choices for patients and administer direct treatment [38]. Clinical outcomes, such as blood pressure control, have significantly improved for patients using telemedicine for hypertension therapy, and these improvements are at par with those of patients receiving personal attention.

In addition to telemonitoring, our study revealed that teleconsultation is a commonly employed strategy in primary care for the management of hypertension. For many patients seeking primary care, teleconsultation is a successful substitute for in-person consultation [60]. Transfers to central clinics may decline as a result of teleconsultations between distant physicians and non-physicians [61]. In rural communities, teleconsulting services enhance primary service compliance and secondary service integration.

Our results demonstrate that asynchronous technology is the most common type of hypertension control technology used in primary healthcare. Asynchronous telemedicine is used by the majority of telemedicine systems of the most recent generation. This technology uses established communication protocols, is easy to use, has stable connections, is dependable, and has considerable bandwidth. For instance, mHealth and eHealth are steadily taking on important roles in the treatment of hypertension patients. Java client apps that send structured XML text and digital camera images as emails are made to be used in underdeveloped nations with limited resources and inadequate networks [62]. By allowing for expert consultation from distant places, the use of cutting-edge technology in rural medical facilities may reduce the cost of managed care insurance policies and patient treatment. Asynchronous telehealth can improve patient and provider satisfaction, reduce unnecessary referrals, and lessen wait times.

According to our findings, telehealth is presently being used in primary healthcare to assist in the management of hypertension. It also makes blood pressure monitoring possible and aids in giving medical practitioners remote access. During the COVID-19 pandemic, it was utilized more frequently to guarantee continuity of care and enhance access to medical services. Research has demonstrated the efficacy of telehealth interventions, such as team-based care and remote patient monitoring, in the treatment of patients with cardiovascular disorders and hypertension. Patients with chronic kidney disease who have hard-to-control hypertension have also shown improvement thanks to a joint nephrologist-pharmacist telemedicine clinic [63,64]. It has been demonstrated that telehealth-delivered strategies, including the TEAM intervention, enhance the management of hypertension and blood pressure. The use of telemedicine to treat non-communicable disorders such as hypertension has increased since the COVID-19 pandemic. Telehealth is a useful tool in the treatment of hypertension because it can, in general, lower obstacles to healthcare access, enhance clinical outcomes, and expand services to remote locations [65].

Healthcare and health systems vary from nation to nation. Notwithstanding these variations, the majority of health systems strive to enhance patient well-being, respond to patient demands, and maintain financial viability [66]. Differences in general health conditions result from the greater difficulty of developing countries in establishing robust and dependable health systems in comparison to wealthy nations [67]. Socioeconomic and cultural disparities are the main causes of health disparities that exist both within and between nations.

Our results demonstrate that various nations employ various technologies to treat hypertension. Developed and underdeveloped nations have different access to telehealth and eHealth technologies. Particularly during the COVID-19 epidemic, telemedicine and eHealth services have been used more frequently in affluent nations [68,69]. Specific telehealth provision benchmarks have been met by these nations, such as a specific degree of telecommunication availability, a percentage of elderly people over 10%, or a percentage of health spending reaching 3-5% of the GDP [70]. Though the concepts and strategies differ according to the degree of development and the government’s commitment to offering reasonably priced healthcare services, developing nations are also using telehealth technology [71].

## Conclusions

Healthcare providers believe that telehealth holds promise for managing hypertension. According to the findings of this review, telemonitoring is the most used telehealth strategy for managing hypertension in primary healthcare. In asynchronous telehealth, this technique is more prevalent. However, the effectiveness of telemedicine services in assisting primary care physicians in managing hypertension requires more research.
